# A Strong Candidate Gene for Nonsyndromic Intellectual Disability Phenotype: *SGSM3*

**DOI:** 10.1111/cge.14631

**Published:** 2024-10-10

**Authors:** Ayberk Turkyilmaz, Kubra Adanur Saglam, Mustafa Yilmaz, Alper Han Cebi

**Affiliations:** ^1^ Department of Medical Genetics, Faculty of Medicine Karadeniz Technical University Trabzon Turkey

**Keywords:** candidate gene, intellectual disability, novel phenotype, SGSM3

## Abstract

SGSM proteins are small modulator proteins interacting with proteins in the RAS signaling pathway. Studies with mouse and human tissues indicated that SGSM genes were highly expressed in the brain and could be expressed at different levels at different stages of development in fetal and adult brain tissue. It was first reported by Birnbaum et al. that the *SGSM3* gene might be associated with a Mendelian inherited disease in families of Ashkenazi Jews with clinical manifestations of intellectual disability (ID). In this study, a novel homozygous stop‐gain (NM_015705.6: c.1576C>T: p.(Arg526Ter)) variation was detected in the *SGSM3* gene in two siblings with short stature and ID findings. The report of two cases with bi‐allelic LOF variants in the *SGSM3* gene from different populations with similar clinical manifestations strengthens the potential of this gene as a candidate gene for the nonsyndromic ID phenotype. Functional studies are required to investigate the signaling pathways affected by *SGSM3* gene variations to produce the ID phenotype and their effect on the functioning of neurons.

## Graphical abstract

*SGSM3* loss‐of‐function cause nonsyndromic intellectual disability phenotype.

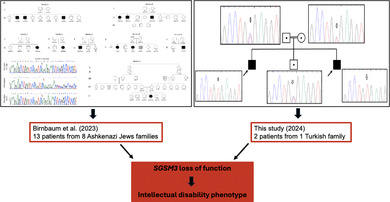

## 1 Introduction

Small G proteins are molecules in the RAS superfamily involved in cell signal transduction and vesicular transport, depending on the exchange between guanosine triphosphate‐bound active and guanosine diphosphate‐bound inactive forms [1]. RAP subfamily proteins of the RAS superfamily are involved in integrin‐mediated cell adhesion, cadherin‐mediated cell junction formation, actin‐mediated cell motility, neuronal cell differentiation, and synaptic plasticity [2, 3, 4, 5, 6, 7]. On the other hand, RAB subfamily proteins of the RAS superfamily are involved in vesicle formation, motility, fusion, and synaptic transmission, as well as in establishing connections between the plasma membrane and Golgi in the cell [8, 9, 10]. The small G protein signaling modulator (SGSM) gene family was reported by Yang et al. and consists of three different members (SGSM1/2/3) [11]. It is well‐established that each of the proteins encoded by *SGSM* genes contains TBC and RUN domains, through which they interact with RAB and RAP proteins, respectively [11]. It was suggested that *SGSM* genes might serve as modulators of RAP‐ and RAB‐mediated cell signaling pathways through this interaction [11].

Lee et al. reported that merlin‐associated protein (MAP), which consisted of 749 amino acids and had a molecular weight of 84.5 kDa, was identical to the protein encoded by the *SGSM3* gene [12]. SGSM3 protein contains a TBC domain in the N‐terminal region, a RUN domain in the C‐terminal region, and an SH3 domain between these domains, which is not observed in other SGSM proteins [12]. Accordingly, Lee et al. suggested that MAP protein might be an adaptor protein involved in protein–protein interaction between signaling molecules [12]. It was shown that SGSM3 (or MAP) protein was expressed in several human and mouse tissues [11, 12]. Studies with mouse and human tissues indicated that *SGSM* genes were highly expressed in the brain and could be expressed at different levels at different stages of development in fetal and adult brain tissue [11]. The results of the previous studies on neuronal cells suggested that SGSM proteins had a strong association with neuronal functions, might assume a function downstream of the RAP signaling pathway, and regulate vesicle traffic through interaction with RAB proteins [11].

It was first reported by Birnbaum et al. that the *SGSM3* gene might be associated with a Mendelian inherited disease in eight different families of Ashkenazi Jews with clinical manifestations of intellectual disability (ID) [13]. In all 13 index cases, the *SGSM*3 gene included the same homozygous frameshift variant (NM_015705.6: c.981dup, p.Glu328ArgfsTer90), and it was suggested that this variant might be the founder variant for the Ashkenazi Jewish population [13]. This study suggests that the *SGSM3* gene is a candidate gene for the nonsyndromic ID phenotype. Notwithstanding the above, this suggestion should be studied in larger patient cohorts to investigate the phenotype, and the pathogenesis of the disease should be investigated using functional studies [13].

This study aimed to present two Turkish siblings with clinical manifestations of ID who had a novel homozygous stop‐gain variant in the *SGSM3* gene, which was previously reported in the literature as a candidate gene for the nonsyndromic ID phenotype.

## 2 Materials and Methods

The probands and their families gave their written informed consent to participate in this study and for genetic analysis. The proband was evaluated by a clinical geneticist. For whole‐exome sequencing (WES) analysis, genomic DNA was isolated from peripheral blood using the QIAamp DNA Blood Mini QIAcube Kit (Qiagen, Hilden, Germany) as per the manufacturer's instructions. All coding regions in the human genome were sequenced to 150 bp at both ends (pair‐end) on the Illumina NovaSeq Platform using the Agilent SureSelect V5 kit (Agilent, Santa Clara, CA, USA). The raw data were analyzed with the Qiagen Clinical Insight data analysis platform. Variant classification was done according to the guidelines of the American College of Medical Genetics and Genomics (ACMG) [14].

## 3 Results

### 3.1 Patient 1

A 36‐year‐old male patient was the first child of consanguineous parents (first cousin marriage) with a previous unremarkable prenatal and natal history. Upon review of the developmental stages of the case, it was understood that the patient started walking at the age of 2 years, had poor academic success, and was not able to read and write. The patient with known aggressive behavior and an irritable personality, was being followed up upon diagnosis of hypertension for 2 years. The height and head circumference were 164 cm (−1.98 standard deviation score [SDS]) and 56 cm (−1.13 SDS), respectively. Dysmorphology assessment indicated no significant findings except prominent ears. The patient had grade 1 hepatosteatosis and left ventricular hypertrophy, with unremarkable brain magnetic resonance imaging (MRI). Hearing and eye examinations were unremarkable.

### 3.2 Patient 2

A 17‐year‐old male patient was the brother of the first case and had no remarkable prenatal and natal history. The birth weight of the patient was 2900 g C/S. Upon review of the developmental stages of the case, it was understood that the patient started to sit without support at the age of 8 months, started to walk at the age of 2 years, and started to speak single words at the age of 2 years. The height and head circumference were 163 cm (−1.85 SDS) and 57 cm (−0.33 SDS), respectively. Dysmorphology assessment indicated low‐set ears and dolichocephaly. Abdominal ultrasonography, echocardiography, and brain MRI were not remarkable. Hearing and eye examinations were unremarkable.

**FIGURE 1:**
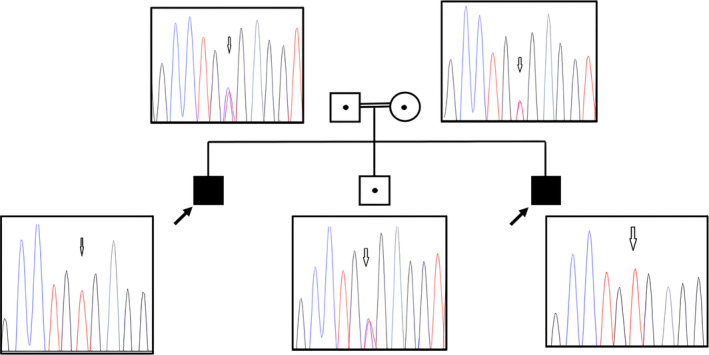
The affected siblings were homozygous for NM_015705.6: C.1576C>T: P.(Arg526Ter) variant in *SGSM3* gene. His healthy brother and parents were heterozygous for this variant.

Karyotype and chromosomal microarray analyses of both patients were normal. Initial WES analysis of Patient 1 did not detect any variants in an OMIM gene known to have been related to ID phenotypes. Pedigree analysis suggested that possible autosomal recessive or X‐linked inheritance might account for the phenotype of the cases. In the second stage, WES analysis was performed on the other affected sibling (Patient 2), and the next‐generation sequencing data of both patients were assessed together. In particular, variations compatible with autosomal recessive and X‐linked inheritance were duly analyzed. The following criteria were applied in the filtering stage: (i) variants with a frequency below 1% in population databases, (ii) variants with a frequency below 1% in the in‐house database (WES data of 3000 Turkish cases studied on the same genetic analysis platform), (iii) pathogenic/likely pathogenic/variant of uncertain significance variants based on the ACMG criteria, and (iv) homozygous/hemizygous variants in both siblings. Upon analysis based on the above criteria, three variants compatible with autosomal recessive inheritance (*SGSM3*, *XPNPEP3*, and *TTLL1*) and one variant compatible with X‐linked inheritance (*RTL9*) were found. It was concluded upon assessment of the variants in the context of segregation analysis, in silico analyses, gene function/expression, and previous literature data, that the stop‐gain variant in the *SGSM3* gene (Chr22: 40803844C>T [hg19]; NM_015705.6: c.1576C>T: p.(Arg526Ter)) might be the top candidate variant [13]. This variation with a global allele frequency of 0.0003105% in gnomAD (v4.1.0) was located in exon 14 and was never reported in the in‐house database. Furthermore, there was no case reported with a homozygous variant in the *SGSM3* gene in the in‐house database. This variation was considered likely pathogenic (PVS1_Moderate: 2 points, PM2_Supporting: 1 point, PP1_Supporting: 1 point, and PM3_Moderate: 2 points) based on the ACMG criteria (total score: 6 points). This variant was heterozygous in healthy parents and the brother (Figure 1).

## 4 Discussion

SGSM proteins are small modulator proteins interacting with proteins in the RAS signaling pathway [11]. They are expressed in several tissues, especially the central nervous system [11]. It has been suggested that they likely play a role in cell signal transduction and vesicular transport through interaction with RAP and RAB proteins in the subfamily of the RAS signaling pathway [11]. It is well‐established that genetic defects in molecules involved in the RAS signaling pathway are associated with short stature, neurodevelopmental delay, neurodegenerative diseases, cancer, and immunodeficiency phenotypes [15, 16, 17]. Upon a review of our cases and the patients previously reported in the literature by Birnbaum et al. together, most of the patients had mild to moderate ID, short stature, mild dysmorphic features, and behavioral abnormalities. All cases with bi‐allelic loss‐of‐function (LOF) variants in the *SGSM3* gene had clinical similarities with phenotypes reported for RAS signaling pathway defects. Nevertheless, the fact that the patients with variants in the *SGSM3* gene had mild facial dysmorphic manifestations and that other organ anomalies did not frequently concur with the ID phenotype suggests that it is associated with a milder clinical picture.

Previous studies on the *SGSM3* gene in mouse and human tissues suggested that this gene might assume important functions in the central nervous system through interaction with RAP and RAB proteins [11]. So far, no bi‐allelic LOF variants for the *SGSM3* gene have been reported in gnomAD (v4.1.0) and our in‐house database. Similarly, there are no bi‐allelic LOF variants in *SGSM1* and *SGSM2* genes in our in‐house database. Upon analysis in the light of all aforementioned data, the following factors were suggestive of the fact that the *SGSM3* gene was a strong candidate gene for the nonsyndromic ID phenotype: (i) no homozygous LOF variant in *SGSM3* gene was previously reported in healthy population databases; (ii) cell culture studies reported that it might assume important functions in the central nervous system through interaction with RAP and RAB proteins; (iii) nonsyndromic ID phenotype with similar clinical manifestations was reported in 15 cases from a total of nine families in two different populations (Turkish and Ashkenazi Jews); and (iv) the candidate variant co‐segregated with the disease in family studies.

**FIGURE 2:**
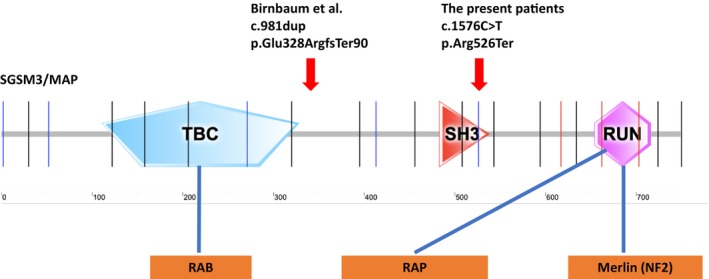
The schematic presentation of the SGSM3 protein. Variations detected in the previous article and in our cases are shown in the upper part of the figure. The bottom part of the figure shows that the SGSM3 protein interacts with various proteins through its different domains.

The first of the variants reported so far as pathogenic in the *SGSM3* gene is the NM_015705.6: c.981dup, p.Glu328ArgfsTer90 variant in 13 cases from a total of eight families of Ashkenazi Jews, and the second is the NM_015705.6: c.1576C>T, p.(Arg526Ter) variation reported in two sibling cases from a family of Turkish origin. Both variations are predicted to induce premature stop codon formation, leading to truncated protein formation and/or nonsense‐mediated decay. The variations in question are associated with the lack of RUN domain in the SGSM3 truncated protein (Figure 2). This may disrupt the interaction with RAP proteins provided via the RUN domain and affect the motility, differentiation, and cell polarity of neurons. In case variations lead to nonsense‐mediated decay, the interaction with RAB proteins may be disrupted due to the lack of other domains of the protein (TBC and SH3 domains), and vesicular transport may be affected. It is also well‐established that SGSM3 protein interacts with merlin protein via its RUN domain, and merlin protein is a negative regulator of the RAS signaling pathway. Lack of the RUN domain may also disrupt the interaction with the merlin protein, leading to impaired negative regulation of the RAS signaling pathway.

In conclusion, the report of two cases with bi‐allelic LOF variants in the *SGSM3* gene from different populations with similar clinical manifestations strengthens the potential of this gene as a candidate gene for the nonsyndromic ID phenotype. Future studies with large cohorts are required to better understand the clinical manifestations of the *SGSM3* gene‐associated phenotype. Functional studies are required to investigate the signaling pathways affected by *SGSM3* gene variations to produce the ID phenotype and their effect on the functioning of neurons.

## Conflicts of Interest

The authors declare no conflicts of interest.

### Peer Review

The peer review history for this article is available at https://www.webofscience.com/api/gateway/wos/peer‐review/10.1111/cge.14631.

## Data Availability

The data that support the findings of this study are available from the corresponding author upon reasonable request.
